# Determination of risk factors affecting the in-hospital prognosis of patients with acute ST segment elevation myocardial infarction after percutaneous coronary intervention

**DOI:** 10.1186/s12872-017-0660-9

**Published:** 2017-09-12

**Authors:** Rui Wang, Biqi Mei, Xinlong Liao, Xia Lu, Lulu Yan, Man Lin, Yao Zhong, Yili Chen, Tianhui You

**Affiliations:** 1 School of Nursing, Guangdong Pharmaceutical University, Guangzhou, 510310 China; 2 School of Public Health, Guangdong Pharmaceutical University, Guangzhou, 510310 China; 3 Department of Cardiology, The First Affiliated Hospital of Sun Yat-Sen University, Guangzhou, 510080 China

**Keywords:** Acute myocardial infarction, ST segment elevation, Percutaneous coronary intervention, Prognosis, Risk factors

## Abstract

**Background:**

To determine the factors affecting the in-hospital prognosis of patients with acute ST segment elevation myocardial infarction (STEMI) after percutaneous coronary intervention (PCI), and to establish its prognostic discriminant model.

**Methods:**

A total of 701 consecutive STEMI patients undergoing PCI were enrolled in this study. The patients were divided into two groups, good prognosis and poor prognosis, based on whether the patient had adverse outcomes (death or heart function ≥ grade III) at discharge. Demographic and basic clinical characteristics, diagnosis at admission (e.g., ventricular function, complications, or hyperlipidemia), and biomedical indicators (e.g., blood count, basal metabolism and biochemical composition, blood lipid and glucose levels, myocardial biomarkers, and coagulation) were collected and analyzed.

**Results:**

We determined 22 factors as risk factors for the in-hospital prognosis of STEMI patients after PCI: age, cardiac function during hospitalization, complications, history of diabetes mellitus, et al., among which the history of diabetes, uric acid, urea nitrogen, and activated partial thromboplastin time (APTT) were independent risk factors.

**Conclusion:**

We identified four independent risk factors for the in-hospital prognosis of STEMI patients after PCI and generated a prognostic model to predict the adverse outcomes of these patients.

## Background

Cardiovascular diseases are the leading cause of death worldwide (http://www.who.int/), and acute myocardial infarction (AMI) is one of the major cardiovascular diseases that leads to a high mortality and morbidity. Based on the ESC/ACCF/AHA/WHF Expert Consensus Document in 2012 [1], AMI has two major types: ST segment elevation myocardial infarction (STEMI) and non-ST segment elevation acute myocardial infarction (NSTEMI). STEMI and NSTEMI exhibit differences in clinical manifestations, pathology, pathogenesis, treatment, and prognosis. For example, compared with NSTEMI, STEMI has a rapid disease progression and a high short-term mortality, although one study has shown a decline in the magnitude and long-term mortality due to STEMI and NSTEMI in recent years [2]. Percutaneous coronary intervention (PCI), due to its fast and sustained opening of the infarct-related artery, has been the mainstay treatment of STEMI. ESC/AHA has recommended that STEMI patients with chest pain lasting less than 12 h should have an emergency PCI within 90 min [3]. Indeed, if a timely diagnosis and treatment of STEMI patients are carried out with PCI, myocardial tissue perfusion can be quickly resumed, and the relevant collateral circulation can be established, subsequently improving heart function and reducing the mortality [4]. However, various factors, including age, heart rate, and the occurrence of no-reflow and/or slow blood flow after PCI, affect the outcomes of STEMI patients after PCI, and these factors are associated with long-term adverse cardiac events as well [5, 6]. Therefore, to assess the risk factors for STEMI patients accurately, it is critical for us to identify high-risk patients and to execute surgical intervention at an earlier phase, thus reducing the incidence of major adverse cardiac and cerebrovascular events and mortality as well as improving the prognosis of patients.

Prior studies have shown that the death of STEMI patients mainly occurs in the first month after AMI [7] and may be heightened by other factors, including age, serious arrhythmias, cardiogenic shock, or heart failure [7]. Although a number of factors including the baseline characteristics such as age, heart rate, blood pressure, and Killip classification have been shown to be independent predictors of long-term death of AMI patients [8, 9], the factors that may be used to predict the short-term prognosis, i.e., in-hospital outcomes of STEMI patients after PCI, have not been systemically explored. In the present retrospective study, we analyzed the clinical data of 701 STEMI patients undergoing PCI at admission, examined the risk factors for a poor in-hospital prognosis of these patients, and attempted to establish a model that may be used to evaluate the in-hospital prognosis of STEMI patients after PCI.

## Methods

### Patient selection

A total of 701 consecutive STEMI patients who underwent emergency PCI treatment between January 1, 2010 and November 30, 2013 at the Coronary Care Unit of Guangzhou City Hospital were included in this study. All demographic and basic clinical characteristics of the STEMI participants were retrospectively analyzed. This study was approved by the Hospital Ethics Committee. All patients provided written informed consent prior to study participation. The diagnosis of STEMI was based on the standards proposed in the “2010 ST-segment elevation acute myocardial infarction diagnosis and treatment guidelines.”

The main inclusion criteria were as follows: (1) increased cardiac biomarker levels (e.g., troponin), or initially increased levels followed by a decrease, exceeding the 99th percentile of the upper limit at least once, and accompanied by at least one of the following: clinical symptoms of myocardial ischemia; new ST segment changes or left bundle branch block (either STEMI or non-STEMI) revealed by electrocardiogram (ECG); a pathologic Q wave in the ECG; new vitality myocardial loss or regional wall motion abnormalities revealed by imaging examination. (2) Patients with normal baseline troponin levels who underwent PCI and had cardiac biomarkers exceeding the upper limit, suggesting perioperative myocardial necrosis. (3) Patients with normal baseline troponin levels who underwent coronary artery bypass grafting and exhibited increased cardiac biomarker levels greater than the upper limit of normal, suggesting perioperative myocardial necrosis. (4) Patients who exhibited pathological changes indicating the presence of AMI, including coronary atherosclerosis, coronary collateral circulation, myocardial gross specimen changes, and histological and ultrastructural changes. Patients with one of the following were excluded from our study: (1) suspected STEMI that did not meet the above diagnostic criteria; (2) STEMI caused by a thrombus originating from the peripheral circulatory system; (3) STEMI caused by coronary blood flow interruption originating from invasive treatment; (4) STEMI caused by coronary blood flow interruption originating from other diseases; (5) previous STEMI.

After application of the inclusion and exclusion criteria, a total of 701 patients (male, 611 (87.3%); female, 90 (12.7%)), were eventually included in this study, with the patient age ranging from 31 to 90 years old (mean, 60.45 ± 11.05 years old). The average length of hospital stay was 8.44 ± 5.49 days. At admission, 452 people (64.5%) had cardiac function grade I, 190 (27.1%) had grade II, 34 (4.9%) had grade III, and 25 (3.6%) had grade IV.

### Experimental design

Based on whether the patients had adverse outcomes at discharge, they were divided into a good prognosis group (cardiac function grade I or II) or a poor prognosis group (cardiac function grade ≥ III or death). The NYHA functional class according to Killip classification was used to categorize NYHF cardiac function: grade I, no signs of heart failure, but pulmonary capillary wedge pressure may be increased; grade II, mild-to-moderate heart failure, pulmonary rales appear in less than 50% of the lungs, may be accompanied by a third heart sound (i.e., ventricular gallop), persistent sinus tachycardia and other arrhythmias, and have increased venous pressure and pulmonary congestion revealed by X-ray; grade III, severe heart failure, acute pulmonary edema, pulmonary rales appear in more than 50% of the lungs; grade IV, cardiogenic shock, systolic blood pressure (SBP) < 90 mmHg, urine output of less than 20 mL/h, clammy skin, cyanosis, rapid breathing, and pulse rate > 100 beats/min.

### Outcome measures

All participants were analyzed based on the following data: 1) general information, e.g., age, gender, and history of other diseases; 2) admission diagnosis, e.g., cardiac function, complications (arrhythmia, heart failure, cardiogenic shock), hyperlipidemia, hypertension, and diabetes; 3) admission vital signs, e.g., SBP, diastolic blood pressure (DBP), heart rate; 4) biochemical indicators, all of which were the results of the first blood test after emergent admission before any treatment was initiated, thus avoiding the effects of treatments on the results. However, we did not rule out the possible impact of the oral administration of aspirin or nitroglycerin and other drugs that had been used to ease the heart symptoms before admission. The following parameters were analyzed: (i) blood count: white blood cell (WBC) count, neutral leaf granulocyte count, neutral leaf granulocyte proportion, hemoglobin, and platelet count (PLT), all of which were analyzed with a Hitachi 7180 automatic biochemical analyzer (Japan); (ii) basic biochemical composition: potassium (K^+^), sodium (Na^+^), chlorine (Cl^−^), calcium (Ca^2+^), glucose (GLU), creatinine, urea nitrogen, and uric acid, all of which were determined with an Abbott c16000 biochemical analyzer (ARCHITECT-c16000); (iii) blood lipids: total cholesterol (TC), triglyceride (TG), high-density lipoprotein cholesterol (HDL-c), low-density lipoprotein cholesterol (LDL-c), apolipoprotein E (APo-E), lipoprotein a (Lp-a), and high-sensitivity C-reactive protein (Hs-CRP), all of which were determined with a Hitachi 7180 automatic biochemical analyzer; (iv) cardiac biomarkers: creatine kinase (CK-MB), troponin (c-TnT), and brain natriuretic peptide (BNP), all of which were determined with a Roche automatic electric hair chemical light immunity analyzer (COBAS 6000; Germany); (v) coagulation test: prothrombin time (PT), activated partial thromboplastin time (APTT), thrombin time (TT), fibrinogen (Fbg), and D-dimer, all of which were determined with a Sysmex automated coagulation instrument (CS-5100; Japan).

### Quality control of data entry

The double-entry method was used to ensure data accuracy. Data retrieval was performed by the quality control staff. After data collection, professional technicians examined the data accuracy and corrected any errors such as data omission and other issues. All medical records were in compliance with the “Guangdong Province Records Writing” standards.

### Statistical analysis

All statistical analyses were performed using SPSS20.0 statistical software. Patient characteristics were presented as the mean ± standard deviation (M ± SD) for continuous variables and as count (percentage) for dichotomous traits. The chi-squared test was used to determine the significant differences in diagnoses at admission and the biochemical indices between patients in the good and poor prognosis groups. Multivariate analysis with logistic regression was used to determine the potential risk factors. Before performing logistic regression analysis, we performed collinearity diagnosis of all factors to determine the collinearity. The factors related to collinearity were either merged or removed based on our professional knowledge. According to single or multi-factor screening, a Fisher discriminant prognostic model was established. The statistical significance was determined at the level of *P* = 0.05.

## Results

### Determination of independent risk factors for the in-hospital prognosis of STEMI patients after PCI

All the basic and clinical characteristics of the STEMI patients who had undergone PCI are shown in Table 1. First, we used univariate analysis to determine the potential factors that significantly influenced the in-hospital prognosis of the STEMI patients after PCI. As shown in Table 2, age, cardiac function at admission, complications, a history of diabetes mellitus, SBP, DBP, heart rate, CK-MB, WBC count, neutral leaf granulocyte count, the proportion of neutral leaf granulocytes, hemoglobin, K^+^, Na^+^, Cl^−^, Ca^2+^, GLU, creatinine, urea nitrogen, uric acid, PT, and APTT substantially affected the in-hospital prognosis of the STEMI patients after PCI (*p* < 0.05 or *p* < 0.01, respectively). Other factors, such as gender, hyperlipidemia at admission, and a history of hypertension or stroke, did not show any significant link to the in-hospital poor outcomes of STEMI patients after PCI.

**Table 1:** Demographic and basic clinical characteristics of STEMI patients undergoing PCI

Variable	Assigned value
Prognosis	Good = 0; Poor = 1
Gender	Male = 1; Female = 2
Age (years)	< 45 = 0; 45–59 = 1; ≥ 60 = 2
Cardiac function at admission	I = 0; II = 1; III = 2; IV = 3
Hyperlipidemia	Yes = 0; No = 1
Complications	Yes = 0; No = 1
SBP	≤ 90 = 0; 91–139 = 1; ≥ 140 = 2
DBP	< 60 = 0; ≥ 60 = 1
HR	< 75 = 0; 75–94 = 1; ≥ 95 = 2
History of hypertension	Yes = 0; No = 1
History of diabetes	Yes = 0; No = 1
History of stroke	Yes = 0; No = 1
Family medical history	Yes = 0; No = 1
WBC count (×10^9^/L)	< 4 = 0; 4–10 = 1; > 10 = 2
Neutral leaf granulocyte count (×10^9^/L)	< 1.8 = 0; 1.8–6.4 = 1; > 6.4 = 2
Neural leaf granulocyte percentage (%)	< 0.46 = 0; 0.46–0.75 = 1; > 0.75 = 2
Hemoglobin (g/L)	< 120 = 0; 120–160 = 1; > 160 = 2
PLT (×10^9^/L)	< 100 = 0; 100–300 = 1; > 300 = 2
K^+^ (mM)	< 3.5 = 0; 3.5–5.3 = 1; > 5.3 = 2
Na^+^ (mM)	< 135 = 0; 135–145 = 1; > 145 = 2
Cl^−^ (mM)	< 96 = 0; 96–110 = 1; > 110 = 2
Ca^2+^ (mM)	< 2.1 = 0; 2.1–2.6 = 1; > 2.6 = 2
GLU (mM)	≤ 6.0 = 0; > 6.0 = 1
Creatinin (μM)	< 53 = 0; 53–115 = 1; > 115 = 2
Urea nitrogen (mM)	< 2.9 = 0; 2.9–8.6 = 1; > 8.6 = 2
Uric acid (μM)	< 142 = 0; 142–452 = 1; > 452 = 2
TC (mM)	< 3.1 = 0; 3.1–5.7 = 1; > 5.7 = 2
TG (mM)	≤ 1.7 = 0; > 1.7 = 1
HDL-c (mM)	< 1.09 = 0; 1.09–1.63 = 1; > 1.63 = 2
LDL-c (mM)	< 3.61 = 0; > 3.61 = 1
APo-E (mg/L)	< 27 = 0; 27–45 = 1; > 45 = 2
Lp-a (mg/L)	≤ 300 = 0; > 300 = 1
Hs-CRP (mg/L)	< 0.1 = 0; 0.1–3 = 1; > 3 = 2
CK-MB (ng/mL)	≤ 4.94 = 0; > 4.94 = 1
c-TnT (ng/mL)	≤ 0.1 = 0; > 0.1 = 1
BNP (ng/L)	≤ 84 = 0; > 84 = 1
PT (s)	< 11 = 0; 11–14 = 1; > 14 = 2
APTT (s)	< 25 = 0; 25–35 = 2; > 35
TT (s)	< 14 = 0; 14–21 = 1; > 21 = 2
Fbg (g/L)	< 2 = 0; 2–4 = 1; > 4 = 2
D-Dimer (μg/L)	≤ 300 = 0; > 300 = 1

**Table 2:** Comparison of demographic and basic clinical data at admission of participants in the good and poor prognosis groups

Outcome measures	n	Poor prognosis (n) (%)	χ^2^	*P*
Gender				
Male	611	49 (8.0)	0.973	0.324
Female	90	10 (11.1)		
Age (years)				
< 45	52	4 (7.7)	7.249	0.027*
45–59	276	14 (5.1)		
≥ 60	373	41 (11.0)		
Admission diagnosis				
cardiac function				
Grade I	452	3 (0.7)	601.143	<0.001**
Grade II	190	1 (0.5)		
Grade III	34	32 (94.1)		
Grade IV	25	23 (92.0)		
Hyperlipidemia				
Yes	108	7 (8.8)	0.620	0.431
No	593	52 (11.9)		
Complications				
Yes	137	19 (13.9)	6.426	0.011*
No	560	40 (7.1)		
SBP (mmHg)				
≤ 90	21	1 (4.8)	27.210	<0.001**
91–139	518	37 (7.1)		
≥ 140	153	21 (13.7)		
DBP (mmHg)				
< 60	82	18 (22.0)	21.688	<0.001**
≥ 60	613	41 (6.7)		
HR (beats/min)				
< 75	342	17 (5.0)	34.651	<0.001**
75–94	268	21 (7.8)		
≥ 95	79	20 (25.3)		
Past history and family disease				
Hypertension				
Yes	299	29 (9.7)	1.112	0.292
No	402	30 (7.5)		
Diabetes				
Yes	175	23 (13.1)	6.759	0.009**
No	526	36 (6.8)		
Stroke				
Yes	15	1 (6.7)	0.061	0.805
no	686	58 (8.5)		
Family medical history				
Yes	154	14 (9.1)	0.116	0.733
No	547	45 (8.2)		
MI index				
CK-MB (ng/mL)				
≤ 4.94	354	22 (6.2)	4.362	0.037*
> 4.94	329	35 (10.6)		
c-TnT (ng/mL)				
≤ 0.1	124	13 (10.5)	0.539	0.463
> 0.1	477	40 (8.4)		
BNP (ng/L)				
≤ 84	36	2 (5.6)	0.408	0.523
> 84	593	51 (8.6)		
Blood test				
WBC count (×10^9^/L)				
< 4	8	1 (12.5)	12.749	0.002**
4–10	420	23 (5.5)		
> 10	264	35 (13.3)		
NLG count (×10^9^/L)				
< 1.8	2	0 (0.0)	6.235	0.044*
1.8–6.4	373	23 (6.2)		
> 6.4	315	36 (11.4)		
NLG proportion (%)				
< 0.46	22	4 (18.2)	18.619	<0.001**
0.46–0.75	476	26 (5.5)		
> 0.75	194	29 (14.9)		
Hemoglobin (g/L)				
< 120	123	19 (15.4)	9.200	0.010**
120–160	529	37 (7.0)		
> 160	40	3 (7.5)		
PLT (×10^9^/L)				
< 100	8	1 (12.5)	0.165	0.921
100–300	543	46 (8.5)		
> 300	142	12 (8.5)		
Blood biomedical test				
K^+^ (mM)				
< 3.5	43	5 (11.6)	18.528	<0.001**
3.5–5.3	639	50 (7.8)		
> 5.3	8	4 (50.0)		
Na^+^ (mM)				
< 135	94	12 (16.0)	8.985	0.011*
135–145	584	42 (7.2)		
> 145	12	2 (16.7)		
Cl^−^ (mM)				
< 96	26	7 (26.9)	11.967	0.003**
96–110	650	50 (7.7)		
> 110	11	1 (9.1)		
Ca^2+^ (mM)				
< 2.1	150	20 (13.3)	6.311	0.043*
2.1–2.6	501	36 (7.2)		
> 2.6	5	1 (20.0)		
GLU (mM)				
≤ 6.0	313	18 (5.8)	5.743	0.017*
> 6.0	377	41 (10.9)		
Creatinine (μM)				
< 53	18	3 (16.7)	40.809	<0.001**
53–115	589	34 (5.8)		
> 115	84	22 (26.2)		
Urea nitrogen (mM)				
< 2.9	24	2 (8.3)	57.631	<0.001**
2.9–8.6	591	35 (5.9)		
> 8.6	65	22 (33.8)		
Uric acid (μM)				
< 142	7	1 (14.3)	28.333	<0.001**
142–452	397	22 (5.5)		
> 452	63	16 (25.4)		
TC (mM)				
< 3.1	55	5 (9.1)	0.258	0.879
3.1–5.7	467	35 (7.5)		
> 5.7	102	7 (6.7)		
TG (mM)				
≤ 1.7	460	37 (8.1)	0.780	0.677
> 1.7	165	10 (6.1)		
HDL-c (mM)				
< 1.09	485	37 (7.6)	1.088	0.580
1.09–1.63	127	10 (7.9)		
> 1.63	13	0 (0.0)		
LDL-c (mM)				
< 3.61	462	37 (7.9)	0.765	0.682
> 3.61	162	10 (6.2)		
APo-E (mg/L)				
< 27	379	29 (7.7)	1.939	0.379
27–45	23	0 (0.0)		
> 45	202	16 (7.7)		
Lp-a (mg/L)				
≤ 300	261	24 (9.2)	2.218	0.330
> 300	341	21 (6.2)		
Hs-CRP (mg/L)				
< 0.1	15	0 (0.0)	1.616	0.204
0.1–3	81	8 (9.9)		
> 3	96	8 (8.3)		
Coagulation				
PT (s)				
< 11	102	8 (7.8)	8.572	0.014*
11–14	504	39 (7.7)		
> 14	37	8 (21.6)		
APTT (s)				
< 25	176	10 (5.7)	8.470	0.014*
25–35	396	33 (8.3)		
> 35	70	12 (17.1)		
TT (s)				
< 14	65	5 (7.7)	1.216	0.545
14–21	579	47 (8.1)		
> 21	57	7 (12.3)		
Fbg (g/L)				
< 2	16	1 (6.3)	0.819	0.664
2–4	336	26 (7.7)		
> 4	291	28 (9.6)		
D-Dimer (μg/L)				
≤ 300	473	43 (9.1)	0.858	0.354
> 300	228	16 (7.0)		

*TC* triglyceride; *MI* myocardial infarction; *NLG* neutral leaf granulocyte

*P ≤ 0.05

**P ≤ 0.01

Next, we sought to determine the independent risk factors for the in-hospital prognosis of the STEMI patients after PCI. The above-identified 22 factors together with gender, history of hypertension, history of stroke, and PLT were included in the logistic multivariate analysis. Although the last four factors were not identified by the univariate analysis as risk factors, we included them in this analysis based on our professional knowledge, which was that these factors might potentially affect the prognosis of patients. As shown in Table 3, a history of diabetes, uric acid, urea nitrogen, and APTT were independent risk factors (*p* < 0.05). If uric acid, urea nitrogen, and APTT were excluded from the reference group, other factors did not show any significant association with the in-hospital prognosis of the STEMI patients after PCI.

**Table 3:** Multivariate logistic regression analysis of independent risk factors for the in-hospital prognosis of STEMI patients after PCI

Relevant factor	*OR* (95% *CI*)	*P*
History of diabetes	2.33 (1.01, 5.36)	0.047
Uric acid (μM)		
142–452 (normal)	1 (reference)	
< 142	0.90 (0.71, 1.15)	0.089
> 452	1.61 (1.04, 2.10)	0.021
Urea nitrogen (mM)		
2.9–8.6 (normal)	1 (reference)	
< 2.9	0.92 (0.68, 0.99)	0.041
> 8.6	5.65 (1.32, 30.40)	0.036
APTT (s)		
96–110 (normal)	1 (reference)	
< 96	0.58 (0.05, 6.25)	0.649
> 110	2.32 (1.31, 15.36)	0.004

### Development and validation of the prognostic prediction model

Since the four independent risk or protective factors identified by the logistic multivariate analysis were categorical variables that were not normally distributed, we used the Fisher discrimination method to generate the prediction model. The eventual Fisher discriminant function was as follows: *Y* = 0.624*X*_1_ + 0.659*X*_2_ + 1.988*X*_3_ + 1.419*X*_4_–4.431 (Table 4). The validation of the discriminant model is shown in Table 5. Basically, the consistency of the discriminant function was 83.9%, the sensitivity was 87.2%, and the specificity was 47.5%.

**Table 4:** Establishment of the Fisher discriminant function model

Prognostic factor	Coefficient
History of diabetes (*X*_1_)	0.624
Uric acid (μM) (*X*_2_)	0.659
Urea nitrogen (mM) (*X*_3_)	1.988
APTT (s) (*X*_4_)	1.419
Constant	−4.431

**Table 5:** Validation of the Fisher discriminant function model

Predictive value N (%)	Original value, n (%)		Total
	Good prognosis	Poor prognosis	
Good prognosis	560 (87.2)	31 (52.5)	591
Poor prognosis	82 (12.8)	28 (47.5)	110
Total	642 (100.0)	59 (100.0)	701

## Discussion

### Effects of age and gender on the in-hospital prognosis of STEMI patients after PCI

In the present study, we showed that gender was not a significant factor that affected the in-hospital prognosis of STEMI patients after PCI. Previous studies have shown that STEMI females have a higher in-hospital mortality than STEMI males [10, 11]. The mechanisms underlying the discrepant results between these studies and ours are probably due to the fact that in these previous studies, the STEMI females were significantly older than the STEMI males, while the ages between these populations were comparable in the present study. Also, the rates of complications and having other diseases were also higher in women than in men in the previous reports. In the present study, we showed that age was positively associated with the prognosis of STEMI patients after PCI, which was consistent with previous reports [12, 13]. It is understandable that aging patients exhibit existing pathological changes in some tissues/organs, which potentially exaggerate the progression of the disease, thus causing direct or indirect adverse impacts on the prognosis of patients after PCI [14, 15]. However, age was not an independent risk factor for the in-hospital prognosis of these STEMI patients. One of the reasons potentially contributing to these different findings was the definition of aging. Previous studies have defined elderly patients as those over 75 years old [16], while our study defined elderly patients as those over 60 years old.

### Effects of heart function at admission and complications on the in-hospital prognosis of STEMI patients after PCI

Our study showed that STEMI patients with cardiac function classification of stage III or IV had a poor prognosis, compared to those with stage I or II classification. In addition, patients who had arrhythmia or cardiogenic shock had a poor prognosis, compared to those without these complications. After myocardial infarction, persistent coronary ischemia can exacerbate the symptoms of heart dysfunction, resulting in a decrease in the left ventricular ejection fraction, which subsequently causes dysfunctions of other organs such as the kidney and brain, eventually giving rise to a poor prognosis [17]. In addition, severe heart dysfunction increases cardiac afterload, which heightens myocardial ischemia and expands the myocardial infarct size, thereby leading to a poor prognosis after PCI in STEMI patients. Arrhythmia including ventricular and atrial fibrillation is a common clinical complication of AMI and is the major factor leading to death of AMI patients [18, 19]. STEMI patients with arrhythmias may develop severe cardiac dysfunction or even sudden cardiac death, thus exerting detrimental impacts on the short- and long-term prognosis of patients after PCI [20, 21]. Therefore, to improve their prognosis, STEMI patients should have control over their arrhythmia before undergoing PCI. Cardiac shock is usually the leading cause of death in AMI patients during hospitalization, often due to a large area of myocardial necrosis [22, 23]. However, our study did not reveal cardiac function at admission or complications as an independent risk factor for the in-hospital prognosis of STEMI patients after PCI.

### Effects of history of diabetes and blood glucose levels at admission on the in-hospital prognosis of STEMI patients after PCI

Our study also showed that a history of diabetes was an independent risk factor for adverse outcomes in AMI patients. It is well known that a history of diabetes is a risk factor for adverse cardiovascular events in AMI patients during hospitalizations [24, 25]. Studies have shown that AMI patients with diabetes have a high ratio of multivessel disease as well as more severe coronary artery stenosis and cardiac dysfunction [26, 27], all of which exacerbate the microcirculation blockade, impair establishment of the collateral circulation, and thus negatively affect the prognosis. In addition, a high blood sugar level in STEMI patients at admission, regardless of the presence of diabetes, has been shown to be closely associated with an increased mortality [28–32], and this association is more pronounced in patients without previous abnormal glucose metabolism [33]. Moreover, our study showed that the blood sugar level in STEMI patients at admission was closely correlated with a poor prognosis after PCI. For patients with no history of diabetes, hyperglycemia not only reflected the disease severity but also promoted endothelial damage, causing vasoconstriction or even narrowing, eventually leading to thrombosis [34]. Therefore, we should closely monitor and control the blood glucose level of STEMI patients, especially in nondiabetic patients, to maximize the benefit of PCI to STEMI patients.

### Effects of admission vital signs on the in-hospital prognosis of STEMI patients after PCI

Epidemiological evidence has shown that heart rate on admission is an independent risk factor in patients with acute myocardial infarction undergoing primary PCI [35, 36]. The increase in heart rate indicates the degree of sympathetic activation in patients with acute myocardial infarction, which in turn increases the oxygen consumption of the myocardium and the myocardial work as well as accelerates the expansion and extension of the myocardial infarction area [37, 38]. The above changes have adverse effects on the structure and function of the heart, leading to the occurrence of heart failure, cardiogenic shock, sudden death, and other adverse cardiovascular events [39]. At present, there are no specific numerical data defining the association of heat rate with the incidence of adverse outcomes of patients with acute myocardial infarction. Our study showed that when the heart rate on admission was more than 95 times/min, the incidence of an adverse prognosis was 25.3%, which was significantly higher than that with the heart rate < 75 times/min (5%) and the heart rate between 75 and 94 times/min (7.8%). Our finding was in line with a previous report [40]. In addition, the blood pressure on admission was also shown to be a risk factor for the in-hospital prognosis of patients with STEMI. The blood pressure on admission was a good indicator of the range of myocardial infarction and the prognosis of patients with acute myocardial infarction [41]. However, the relationship between blood pressure and the prognosis of patients with acute myocardial is not clear. Some studies have suggested that patients with AMI with high blood pressure have a poor prognosis [42], while others have shown that patients with AMI with low SBP and low DBP levels on admission were significantly associated with the risk of in-hospital death [43, 44]. In the present study, we found that the risk factors for the in-hospital prognosis of patients with STEMI included SBP (≥140 mmHg) and DBP (<60 mmHg) on admission. We believe that a higher SBP and/or a lower DBP can affect myocardial perfusion, which will adversely affect the prognosis. On the other hand, the blood pressure may impact the blood lipid levels and inflammatory responses, both of which can synergistically promote the occurrence and development of cardiac insufficiency, leading to adverse outcomes such as heart failure, arrhythmia, or death. Collectively, compared to other clinical and laboratory parameters, the heart rate and blood pressure on admission are the two easiest parameters to collect and measure accurately. By observing these two vital signs, medical staff can determine the severity of myocardial infarction, guide clinical treatment, and appraise the prognosis.

### Association of blood count components at admission with the in-hospital prognosis of STEMI patients after PCI

Our study showed that the WBC count, neutral leaf granulocyte cell count, granulocyte proportion, and hemoglobin levels, but not PLT levels, at admission, had significant impacts on the in-hospital prognosis after PCI in patients with STEMI, but they did not function independently. In recent years, several studies have shown that an increase in the WBC count is an independent risk factor that affects the prognosis of STEMI patients and is closely correlated with cardiac dysfunction and mortality after AMI [45, 46]. An increase in the WBC count in STEMI patients was partially attributed to the aseptic inflammation caused by coronary plaque rupture, and a large number of WBCs infiltrated and accumulated in the ischemic area, leading to clogged capillaries, all of which subsequently produced a variety of neurotransmitters and reactive oxygen species, resulting in microvascular reflow disorders [47–49]. Thus, the WBC levels of patients monitored at admission and after PCI can effectively predict the in-hospital prognosis. The PLT count has been shown to be associated with the severity of coronary artery disease and the prognosis of coronary heart disease patients [50]. AMI may induce platelet adhesion and aggregation to the site of atherosclerotic plaque rupture and erosion, forming blood clots, thereby exacerbating myocardial ischemia [51]. In addition, platelet activation can release serotonin and thromboxane, which in turn strengthen or expand platelet aggregation, further aggravating myocardial infarction [52]. However, our study did not conclude that the PLT count was an independent risk factor, probably due to the fact that our patients underwent primary PCI, which diminished the adverse effects of an abnormal PLT count on the cardiovascular system. In other words, the relative short time of ischemia may reduce the impact of the PLT count on the prognosis.

Sabatine et al. [53] have shown that lower hemoglobin levels at admission were associated with an adverse cardiovascular prognosis of STEMI patients as evidenced by the finding that starting from 14 g/dL, every 1 g/dL decrease in the hemoglobin level significantly increased the mortality. Our results were in line with the above observation. Low hemoglobin levels damage the oxygen supply to the infarct area in AMI patients, increase the incidence of arrhythmias, lower blood pressure, expand the infarct size, and eventually accentuate ventricular remodeling [54]. The high mortality of STEMI patients with anemia was also related to bleeding events. Therefore, some treatments such as antiplatelet, anticoagulant, and thrombolytic therapy as well as coronary revascularization application have been shown to increase the risk of bleeding and worsen anemia, and bivalent anti-platelet drug therapy compared with monotherapy also increases the risk of bleeding complications [55, 56].

### Effects of creatinine, urea nitrogen, and uric acid levels on the in-hospital prognosis of STEMI patients after PCI

Previous studies have shown that renal dysfunction in STEMI patients is one of the most important predictors for the in-hospital and long-term mortality [57] and that the serum creatinine level is closely related to the prognosis after treatment [3, 58, 59]. Our study showed that the two most important indicators of renal function, elevated levels of creatinine and urea nitrogen, significantly affected the in-hospital prognosis of STEMI patients after PCI and that an elevated blood urea nitrogen level was an independent risk factor, probably due to the fact that a high serum creatinine level was associated with an advanced age, poor renal reserve capacity, high blood pressure, and/or diabetes. Early restoration of effective myocardial reperfusion in STEMI patients was the key to reduce acute mortality and improve the prognosis; however, the interventional or drug therapy was often limited by the renal function and serum creatinine levels. Therefore, to develop individualized treatment programs, serum creatinine and urea nitrogen levels should be considered as important predictors of prognosis, and risk stratifications should be conducted based on the functional status of the kidneys as well as the blood urea nitrogen and creatinine levels so as to eventually effectively reduce mortality and improve the in-hospital prognosis.

Numerous studies have demonstrated that a high serum uric acid level is an independent risk factor for adverse cardiovascular events in AMI patients [60, 61]. Indeed, an increase in the uric acid concentration is tightly correlated with a number of coronary heart disease risk factors, including age, sex, history of diabetes, and renal insufficiency [62]; moreover, a high serum uric acid level promotes the formation of blood clots, enlarges the myocardial infarct area, or increases the recurrence of myocardial infarction, leading to adverse cardiac events and a poor prognosis [63, 64]. Therefore, high circulating levels of uric acid should be viewed as a predictor for the in-hospital mortality of AMI patients as previously suggested [65], although it is usually not considered [66]. Our study supported the premise that high uric acid levels at admission, as an independent risk factor, increased the incidence of a poor in-hospital prognosis of STEMI patients after PCI. Therefore, uric acid levels should be monitored in STEMI patients before and after PCI.

### Effects of K^+^/Na^+^ concentration, ATPP, and CK-MB on the in-hospital prognosis of STEMI patients after PCI

Our study showed that either an increase or decrease in the concentration of K^+^ or Na^+^ indicated a poor in-hospital prognosis of STEMI patients after PCI. Particularly, a significantly high concentration of K^+^ markedly increased the incidence of poor outcomes. Previously, hyponatremia has been shown to be one of the indicators for a poor prognosis of AMI patients [67, 68] and has been linked to the myocardial infarct size [69]. Our data suggested that a certain range of Na^+^ concentrations should be maintained in STEMI patients after PCI; either an increase or a decrease beyond this range will predict a poor in-hospital prognosis of STEMI patients.

APTT reflects the functionality of the endogenous coagulation system, is a potential hypercoagulable state marker, and has been reported to be a predictor of coronary heart disease risk [70]. Consistent with this previous report, we observed that APTT was an independent risk factor for the in-hospital prognosis of STEMI patients after PCI, as evidenced by the observation that an elongated PT increased the incidence of a poor prognosis, although the underling mechanisms are not clear. Therefore, monitoring PT and APTT in STEMI patients prior to PCI is important as it can guide the selection of an optimal clinical treatment and predict the prognosis after PCI.

The CK-MB levels may reflect the myocardial infarction size and severity and are also closely associated with the severity of myocardial necrosis, functional recovery of the reperfused region, left ventricular ejection fraction, hospitalization due to congestive heart failure, and mortality 7 days after PCI [71]. In line with the above reports, we also found a positive correlation between the CK-MB level and a poor prognosis of STEMI patients after PCI, while the troponin levels had no effect on the prognosis. Therefore, actively monitoring the concentration of CK-MB in STEMI patients should help determine the diagnosis and prognosis.

### Limitations of our study

Although STEMI patients have standardized diagnostic and treatment guidelines, the evaluation criteria used in this study were simple and practical. However, there were some limitations present in our study that should be noted: (1) significant differences in the baseline characteristics and the treatments of the participants existed; (2) all indicators were measured at admission, i.e. in the acute phase; we did not follow up to verify if these indicators changed over a longer period of time after PCI; (3) we only studied the in-hospital prognosis and did not determine the prognosis over a longer period of time after discharge; and (4) the sample size in our study was relatively small, and it was a single-center study. Therefore, our findings and model should be further corroborated in a large-scale multi-center study in the future.

## Conclusion

In the present study, we demonstrated that the in-hospital prognosis of STEMI patients undergoing emergency PCI is affected by a number of factors and that a history of diabetes, uric acid and urea nitrogen levels, and APTT at admission may act as independent predictors for adverse outcomes during hospitalization. We established a discriminant model for predicting the prognosis of STEMI patients with a consistency of 83.9%, a sensitivity of 87.2%, and a specificity of 47.5%.

## Abbreviations

AMI: Acute myocardial infarction
APo-E: Apolipoprotein E
APTT: Activated partial thromboplastin time
BNP: Brain natriuretic peptide
Ca^2+^: Calcium
CK-MB: Creatine kinase
Cl^**−**^: Countchlorine
c-TnT: Troponin
DBP: Diastolic blood pressure
ECG: Electrocardiogram
Fbg: Fibrinogen
GLU: Glucose
HDL-c: High-density lipoprotein cholesterol
HR: Heart rate
Hs-CRP: High-sensitivity C-reactive protein
K: Potassium
LDL-c: Low-density lipoprotein cholesterol
Lp-a: Lipoprotein a
Na^**+**^: Sodium
NSTEMI: Non-ST segment elevation acute myocardial infarction
PCI: Percutaneous coronary intervention
PLT: Platelet
PT: Prothrombin time
SBP: Systolic blood pressure
STEMI: ST segment elevation myocardial infarction
TC: Total cholesterol
TG: Triglyceride
TT: Thrombin time
WBC: White blood cell
